# Expansion of Murine Gammaherpesvirus Latently Infected B Cells Requires T Follicular Help

**DOI:** 10.1371/journal.ppat.1004106

**Published:** 2014-05-01

**Authors:** Christopher M. Collins, Samuel H. Speck

**Affiliations:** Emory Vaccine Center and Department of Microbiology and Immunology, Emory University School of Medicine, Atlanta, Georgia, United States of America; University of California Berkeley, United States of America

## Abstract

X linked lymphoproliferative disease (XLP) is an inherited immunodeficiency resulting from mutations in the gene encoding the slam associated protein (SAP). One of the defining characteristics of XLP is extreme susceptibility to infection with Epstein-Barr virus (EBV), a gammaherpesvirus belonging to the genus *Lymphocryptovirus*, often resulting in fatal infectious mononucleosis (FIM). However, infection of SAP deficient mice with the related Murine gammaherpesvirus 68 (MHV68), a gammaherpesvirus in the genus *Rhadinovirus*, does not recapitulate XLP. Here we show that MHV68 inefficiently establishes latency in B cells in SAP deficient mice due to insufficient CD4 T cell help during the germinal center response. Although MHV68 infected B cells can be found in SAP-deficient mice, significantly fewer of these cells had a germinal center phenotype compared to SAP-sufficient mice. Furthermore, we show that infected germinal center B cells in SAP-deficient mice fail to proliferate. This failure to proliferate resulted in significantly lower viral loads, and likely accounts for the inability of MHV68 to induce a FIM-like syndrome. Finally, inhibiting differentiation of T follicular helper (T_FH_) cells in SAP-sufficient C57Bl/6 mice resulted in decreased B cell latency, and the magnitude of the T_FH_ response directly correlated with the level of infection in B cells. This requirement for CD4 T cell help during the germinal center reaction by MHV68 is in contrast with EBV, which is thought to be capable of bypassing this requirement by expressing viral proteins that mimic signals provided by T_FH_ cells. In conclusion, the outcome of MHV68 infection in mice in the setting of loss of SAP function is distinct from that observed in SAP-deficient patients infected with EBV, and may identify a fundamental difference between the strategies employed by the rhadinoviruses and lymphocryptoviruses to expand B cell latency during the early phase of infection.

## Author Summary

During an immune response, B cells respond to invading pathogens by undergoing massive expansion during the germinal center reaction. This proliferation requires signals from CD4 T cells, with some B cells then maturing into antibody secreting plasma cells, while others mature into memory B cells that may persist for the life of the host. Gammaherpesviruses take advantage of this immune response by infecting B cells, resulting in expansion of the pool of infected cells during the germinal center reaction. The human gammaherpesvirus Epstein-Barr virus (EBV) is thought to be able to accomplish this without the need for CD4 T cell help by expressing viral proteins that mimic signals from CD4 T cells. Here we show in a mouse model of gammaherpesvirus infection that infected B cells require signals from CD4 T cells for proliferation. Since the mouse gammaherpesvirus and EBV belong to different subgroups of gammaherpesviruses, this suggests that these subgroups utilize fundamentally different strategies to expand the pool of infected B cells during the establishment of latency. These different strategies may explain the different outcome of infection by these different subgroups of gammaherpesviruses in the context of defective germinal center responses that result from defective CD4 T cell help.

## Introduction

X-linked lymphoproliferative disease (XLP) is an inherited immune disorder that commonly manifests after Epstein Barr virus (EBV) infection [1], [2]. XLP is characterized by hypogammaglobulinemia, lymphoma and fulminant infectious mononucleosis (FIM) that consists of polyclonal B and CD8 T cell expansion. These lymphocytic expansions infiltrate the liver and bone marrow, often resulting in secondary hemophagocytic lymphohistiocytosis (HLH).

The genetic defect resulting in XLP has been mapped to the *SH2D1A* gene, which encodes the slam associated protein (SAP) [3]–[5]. SAP is an adaptor protein expressed in T, NK, and NKT cells that binds the intracellular domain of several SLAM family receptors [6]. The inability to control EBV infection in XLP patients has been shown to be due in part to the inability of CD8 T cells to recognize EBV infected B cell targets [7]–[10]. This inability to recognize infected B cells is not specific to EBV as SAP-deficient CD8 T cells have been shown to be unable to recognize other viral antigens when presented on B cells [7]. However, SAP-deficient CD8 T cells are fully capable of recognizing these same antigens when presented on non-B cell targets [7]. The inability to recognize and kill B cell targets by SAP-deficient CD8 T cells can be overcome by blocking the SLAM family receptors NTB-A and 2B4 [7], [9], which is consistent with previous work showing that these SLAM family members have inhibitory functions that prevent recognition of B cell targets in the absence of SAP [9], [11], [12].

Since this extreme susceptibility to EBV infection is thought to be due to the B lymphotropic nature of the virus, it is somewhat surprising that XLP patients do not exhibit the same sensitivity to the closely related human herpesvirus 8 (HHV-8, also known as Kaposi's sarcoma associated herpesvirus or KSHV), which also establishes life-long infection in B cells. Both viruses are members of the subfamily Gammaherpesvirinae, but HHV-8 is placed in the genus *Rhadinovirus* whereas EBV belongs to the genus *Lymphocryptovirus* [13]. There has been a single report of an XLP patient that developed HHV-8 mediated HLH, however this patient did not develop FIM and survived HHV-8 infection [14]. This difference in susceptibility of XLP patients to EBV and HHV-8 may simply be due to the lower prevalence of HHV-8 in the general population, combined with the fact that EBV infection usually precedes HHV-8 infection [15]. Alternatively, it may reflect a fundamental difference in the biology of rhadinoviruses and lymphocryptoviruses.

Several groups have created SAP knockout mice to develop a small animal model of XLP [16]–[18]. Studies using these knockout mice have shown that SAP expression in CD4 T cells is required for generating germinal center reactions [6], [19], [20], which has also been confirmed in XLP patients [21]. In the absence of SAP, CD4 T cells are unable to form stable conjugates with B cells, resulting in the inability to provide helper functions to B cells [6]. Because of this inability to generate a germinal center reaction, SAP-deficient mice are unable to generate memory B cells or long lived plasma cells, and thus have severe defects in humoral immunity [2], [19]. Interestingly, deletion of the SLAM receptor Ly108 (known as NTB-A in humans) restores the ability of SAP-deficient mice to form germinal centers [22]. This finding, coupled with the inhibitory function of NTB-A in mediating formation of stable conjugates between CD8 T cells and B cell targets in the absence of SAP [7], suggests that these SLAM receptors require SAP expression in T cells for mediating stable interactions between B cells and either CD4 or CD8 T cells.

The inability of SAP-deficient mice to form germinal centers is of great interest because of the well-delineated role of EBV in driving B cells through the germinal center reaction [23]. During a T dependent germinal center response, CD4 T cells are initially activated by antigen presenting dendritic cells, and these activated CD4 T cells are the precursors of T follicular helper (T_FH_) cells [24]–[26]. This interaction is mediated by integrins and is SAP independent [6]. Once activated, these CD4 T cells migrate to the border between the T cell zone and B cell follicle where they activate and provide helper function to B cells through cognate interactions. Formation of stable conjugates between CD4 T cells and B cells is SAP dependent [6], and ongoing stimulation by B cells is critical for maintenance of the T_FH_ population [27], [28]. Once activated, the B cells can either migrate to the extra-follicular space where they differentiate into short-lived plasmablasts, or they enter the follicle and initiate formation of a germinal center (GC). EBV is thought to be capable of bypassing the requirement for T_FH_ cells by expressing viral proteins that mimic signaling required for GC B cell formation, actively driving B cells through the GC reaction.

The current model of EBV infection is that the virus uses 4 different transcription programs to drive infected naïve B cells through the GC reaction to gain access to the long-lived memory B cell compartment [reviewed in [23]]. According to this model, EBV infects naïve B cells, and the sequential expression of these transcription programs activates resting cells, which then acquire a GC phenotype and ultimately differentiate to a memory B cell phenotype, thereby gaining access to a long-lived, quiescent pool of cells for the virus to remain latent in. EBV is thought to accomplish this by expression of viral proteins that mimic critical signaling events - LMP1 mimics constitutive CD40 signaling [29] whereas LMP2A provides survival signals that recapitulate those provided by the BCR [30]. This suggests EBV may be able to bypass T_FH_ signals normally required for proliferation of GC B cells, allowing EBV infected cells to proliferate in the absence of a GC reaction in XLP patients.

Because the primary site of HHV-8 infection is unknown, very little is known about early events during infection. As such, it is not known if HHV-8 infection of B cells follows a similar path as EBV in driving naïve B cells through a GC reaction to gain access to the memory pool. However, much more is known about the pathogenesis of the closely related rodent gammaherpesvirus, Murine gammaherpesvirus 68 (MHV68). MHV68 is more closely related to HHV-8 than to EBV, and as such is placed in the genus *Rhadinovirus*. MHV68 naturally infects rodents and infection of laboratory strains of mice has been extensively studied as a small animal model of gammaherpesvirus pathogenesis. Similar to EBV, at the peak of infection the majority of MHV68 latently infected B cells have a GC phenotype [31]–[33] and are found in germinal centers [31]. At late times post-infection, MHV68 latency is predominantly maintained in isotype switched memory B cells [34]. While infection of SAP-deficient mice with MHV68 mimics several features of SAP deficiency in humans, XLP is not fully recapitulated [18], [35], [36]. SAP-deficient mice do not develop FIM or succumb to infection, and although persistent virus production has been noted in the lungs [18], the viral load in the spleen is actually lower than that seen in wild type mice [35], [36].

To begin to understand why MHV68 does not recapitulate XLP in SAP-deficient mice, we utilized a transgenic virus that expresses yellow fluorescent protein, allowing identification of infected B cells [31]. We show that during primary infection with MHV68, infected B cells are localized to B cell follicles and can acquire a GC phenotype, but fail to proliferate. We further show that this inability to proliferate is specifically due to the lack of SAP expression in CD4 T cells, indicating that latently infected B cells require CD4 T cell help for proliferation. Finally, we show that inhibiting differentiation of naïve CD4 T cells to T_FH_ cells in wild type mice results in impaired establishment of MHV68 infection. This data shows that MHV68 requires signals from T_FH_ cells for establishment of infection, suggesting that rhadinoviruses differ from lymphocryptoviruses in that they do not actively induce differentiation and proliferation of infected B cells, instead relying on host determinants for transit through the GC reaction to gain access to the memory B cell pool. This differential requirement for signals from T_FH_ cells during the GC response may explain why XLP patients are more susceptible to EBV than HHV-8.

## Results

### Inefficient Establishment of MHV68 B Cell Latency in SAP-Deficient Mice

**Figure 1:**
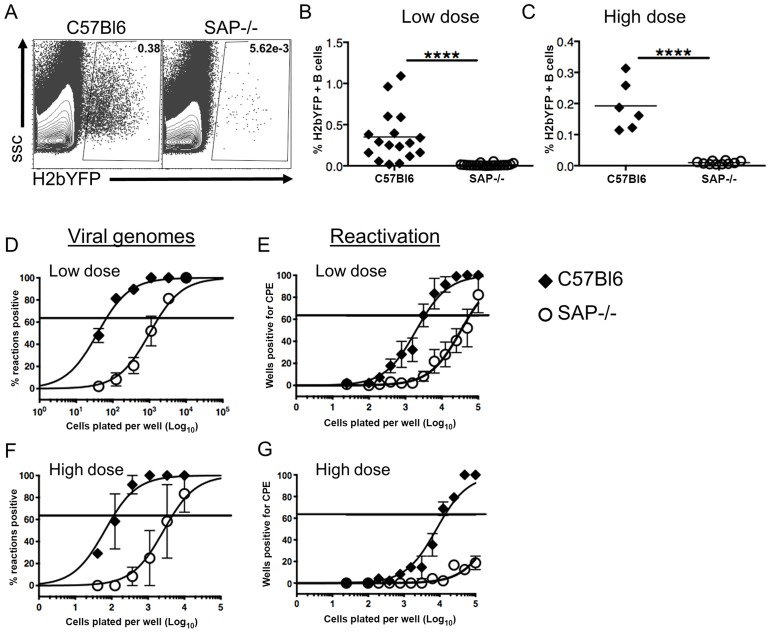
Reduced frequency of latently infected B cells in SAP-deficient mice during the establishment of latency. Mice were infected intranasally with either 1,000(low dose) or 4×10^5^ pfu (high dose) of MHV68-H2bYFP and splenocytes were harvested at days 16–18 post-infection. (A) Representative flow cytometry plots showing identification of virus infected (YFP positive) B cells. Flow plots were gated on CD3−, B220+ cells. (B and C) Frequency of virus infected B cells determined by YFP expression at days 16–18 post-infection following either low dose or high dose infection. Each symbol represents an individual infected mouse, and the horizontal line represents the mean frequency of virus infected B cells. ****, p<0.0001. (D and F) Limiting dilution PCR analysis to determine the frequency of viral genome positive splenocytes. (E and G) Limiting dilution analysis to determine the frequency of infected splenocytes capable of reactivating virus. Serial dilutions of splenocytes were plated on MEF monolayers, and reactivating virus was detected by the presence of cytopathic effect (CPE) 14 days after plating. Results are from 4 (B,D,E) or 2 (C,F,G) independent experiments with 3 to 5 mice per group.

Since previous studies have shown that infection of SAP-deficient mice with MHV68 results in reduced viral load in spleens [35], [36], we infected mice with a recombinant MHV68 that expresses yellow fluorescent protein (MHV68-H2bYFP) [31] to compare the infected B cell populations in these mice with those seen in wild type mice. Mice were infected intranasally with either a low dose (1,000 pfu) or a high dose (4×10^5^ pfu) of virus, and spleens were harvested at 16–18 days post-infection. As shown in figure 1A–C, there were significantly fewer H2bYFP positive B cells in SAP-deficient mice at both doses. Consistent with previously published results [35], [36], there was also a significant reduction in the frequency of viral genome positive cells during the early establishment of latency (Fig. 1D,F). The frequency of viral genome positive splenocytes in wild type mice after low dose infection was 1 out of 66 cells, whereas only 1 out of 1,737 splenocytes contained viral genomes in SAP-deficient mice. The reduced frequency of viral genome positive cells was also reflected in a reduction in the number of splenocytes capable of reactivating virus, as only 1 out of 57,777 splenocytes from SAP-deficient mice were capable of reactivating virus compared to 1 out of 3,387 splenocytes from wild type mice (Fig. 1E).

Similar to the results for low dose infections, the number of genome positive splenocytes from SAP-deficient animals after high dose infection was also significantly lower than in wild type mice. The frequency of viral genome positive cells was 1 out of 136 in wild type mice compared to 1 out of 4,607 in SAP-deficient mice (Fig. 1E). Similarly, there was also a significant defect in the number of cells capable of reactivating virus (Fig. 1F). However, because infection with higher MHV68 doses proceeds with faster kinetics, and contraction of infected cell populations in the spleen occurs earlier, the number of reactivating splenocytes from SAP mice was below the limit of which can be accurately estimated by the limiting dilution reactivation analysis.

### Presence of MHV68 in B Cells with a Germinal Center Phenotype in SAP-Deficient Mice

**Figure 2:**
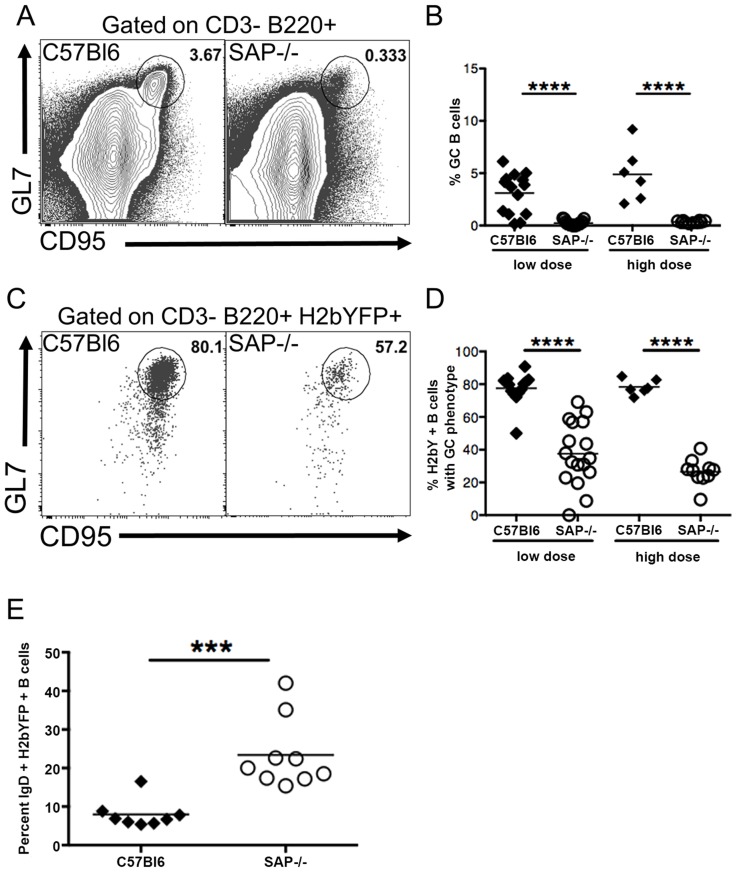
Reduced germinal center response in SAP-deficient mice. Mice were infected with either 1,000(low dose) or 4×10^5^ pfu (high dose) of MHV68-H2bYFP, and splenocytes were harvest at day 16–18 post-infection. (A) Representative flow cytometry plots showing defective germinal center B cell formation in SAP-deficient mice. Flow plots were gated on CD3−, B220+ cells, and germinal center B cells were defined as CD95^HI^, GL7^HI^. (B) Quantitation of the percentage of total B cells that had a germinal center phenotype. Each symbol represents a single infected mouse. ****, p<0.0001. (C) Representative flow plots showing the percentage of infected B cells that had a GC phenotype. Flow plots were gated on CD3−, B220+, H2bYFP+ cells to identify infected B cells, and cells with a GC phenotype were defined as CD95^HI^, GL7^HI^. (D) Quantitation of the percentage of infected B cells that have a GC phenotype. Each symbol represents a single infected mouse. ****, p<0.0001. (E) Percentage of CD3−, B220+, H2bYFP+ cells that express surface IgD after infection with 1,000 pfu of virus and harvested a 16 days post-infection. Each symbol represents a single infected mouse. ***, p = 0.0004. Results in panels B and D were compiled from either 4 (low dose infections) or 2 (high dose infections) independent experiments with 3 to 5 mice per group. Results in panel E were compiled from 2 independent experiments with 4 to 5 mice per group. Each symbol represents a single animal, and the horizontal bar represents the mean.

We next performed flow cytometric analysis of splenocytes to compare the GC B cell populations in SAP-deficient mice with that seen in wild type mice. As expected, SAP-deficient mice exhibited a severe defect in generating a GC response following MHV68 infection (Fig. 2A,B). Interestingly, analysis of the H2bYFP-positive B cell population revealed that although SAP-deficient mice were severely compromised in their ability to generate GC B cells, a significant fraction of infected B cells exhibited a GC phenotype (Fig. 2C,D). However, the overall percentage of infected B cells that had a GC phenotype in SAP-deficient mice was significantly lower than that seen in wild type mice. Since the inability of SAP-deficient mice to generate a GC response has been shown to be due to inefficient CD4 T cell help [37], this data suggests that MHV68 is at least partially dependent on CD4 T cells for differentiating to a GC phenotype. Consistent with this hypothesis, there was a significantly higher percentage of naïve, IgD+ B cells in the H2bYFP positive B cell fraction in SAP-deficient mice (Fig. 2E).

Taken together, this data suggests that MHV68 infects naïve B cells that then are at least partially dependent on CD4 T cell help to differentiate to GC B cells. Initial activation and differentiation of naïve CD4 T cells into T_FH_ precursors is mediated through interaction with dendritic cells and is SAP independent, whereas maintenance of the pool of T_FH_ cells requires continuous stimulation through interaction with B cells, which is SAP dependent [27], [28]. In SAP-deficient mice, this initial differentiation of T_FH_ precursors may be sufficient for differentiation of the limited fraction of infected B cells with a GC phenotype. Alternatively, MHV68 may play a limited role in driving differentiation of infected cells, but CD4 T cell help is required for optimal differentiation.

### MHV68 Infected Plasma Cells Are Not Exclusively Germinal Center Derived

The reduced percentage of infected B cells with a GC phenotype, along with the increased percentage of infected naïve B cells in SAP-deficient mice, suggested that in contrast to what is proposed to happen following EBV infection of B cells [23], MHV68 may not play an active role in activating naïve B cells and driving them through the GC reaction. If MHV68 infected naïve B cells rely on CD4 T cell help for activation and differentiation, then infected extra-follicular short-lived plasma cells should be present at early times post-infection. We have previously shown that a significant percentage of infected splenocytes have a plasma cell phenotype at day 14 post-infection, and this percentage is decreased at day 18 [32]. Although these are likely to be extra-follicular short-lived plasma cells, we cannot exclude the possibility that they arose from the GC pathway since there are no markers useful for differentiating short-lived and long-lived plasma cells. One of the defining characteristics of SAP-deficiency is hypogammaglobulinemia due to the inability to form GCs, and SAP-deficient mice are unable to generate long-lived plasma cells [19]. However, these mice can generate extra-follicular short-lived plasma cells [19]. Therefore, the presence of infected plasma cells in SAP-deficient mice would indicate that, in addition to the follicular pathway, virally infected cells can also enter the extra-follicular pathway, providing evidence that MHV68 does not actively drive infected B cells to the GC pathway.

**Figure 3:**
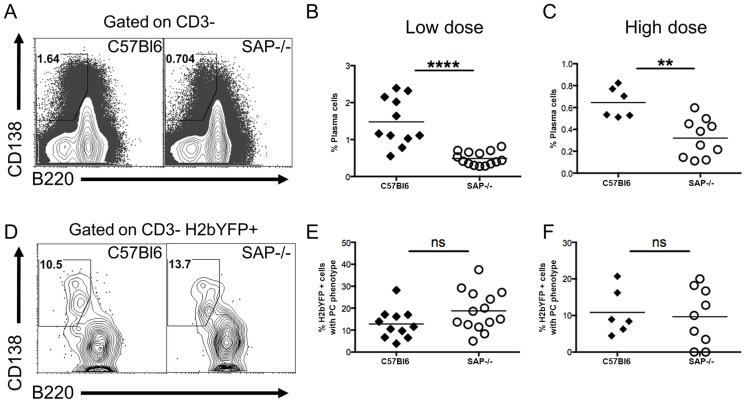
Infected B cells are not restricted to entering the follicular pathway. Mice were infected with either 1,000(low dose) or 4×10^5^ pfu (high dose) of MHV68-H2bYFP, and splenocytes were harvested at days 16–18 post-infection for phenotypic analysis. (A) Representative flow cytometry plots showing plasma cell populations. Flow plots were gated on CD3− splenocytes, and plasma cells were defined as B220^neg-/low^, CD138^hi^. Quantitation of the percentage of plasma cells after infection with either 1,000 pfu (B) or 4×10^5^ pfu (C) of MHV68-H2bYFP. (B & C) Frequency of total splenic plasma cells at days 16–18 post-infection. Each symbol represents a single infected mouse. ****, p<0.0001; **, p = 0.0016. (D) Representative flow cytometry plots showing the percentage of H2bYFP positive cells that have a plasma cell phenotype. Flow plots were gated on CD3− H2bYFP+ cells, and plasma cells are defined as in (A). Quantitation of the percentage of CD3−, H2bYFP+ cells that had a plasma cell phenotype after infection with either 1,000 pfu (E) or 4×10^5^ pfu (F) of MHV68-H2bYFP. Each symbol represents a single infected mouse. The differences between the frequency of virus infected splenocytes exhibiting a plasma cell phenotype was not statistically different between infected C57Bl/6 and SAP−/− mice (panel E, p = 0.09; panel F, p = 0.76). Results were compiled from 4 (panels B & E) or 2 (panels C & F) independent experiments with 3 to 5 mice per group. Each symbol represents a single animal, and the horizontal bar represents the mean.

Analysis of the plasma cell populations at day 16 post-infection showed that although SAP-deficient mice could generate plasma cells, the overall percentage was significantly reduced (Fig. 3A–C). However, this reduction may be the result of the reduced viral load in SAP-deficient mice (see Figs. 1–2A), and not due to a defect in the generation of short-lived plasma cells. Analysis of MHV68 infected cells (H2bYFP-positive population) revealed that there was no difference in the percentage of infected cells that had a plasma cell phenotype in SAP deficient mice compared to wild type mice (Fig. 3D–F). This suggests that a fraction of infected cells can enter the extra-follicular pathway, providing evidence that MHV68 does not play an active role in driving infected B cells to the follicular pathway, but instead is subject to the same fate determination as uninfected B cells.

### MHV68-H2bYFP Infected Cells Localize to B Cell Follicles but Fail to Proliferate

**Figure 4:**
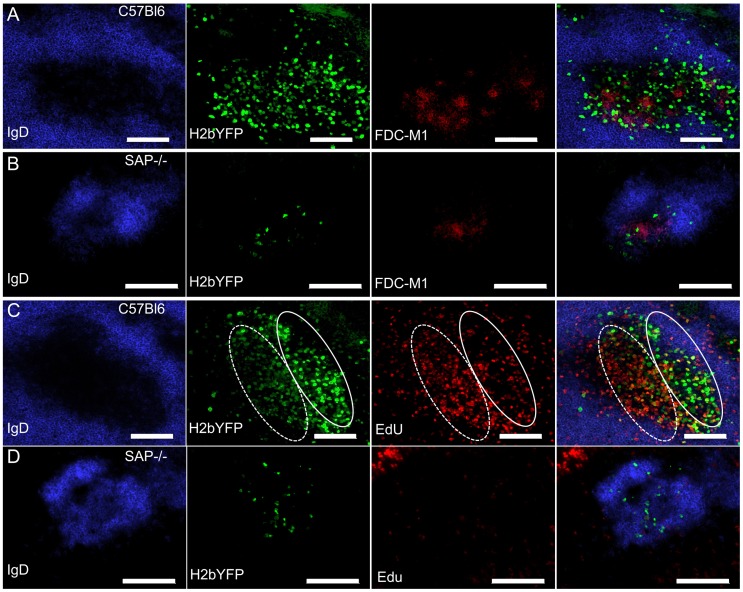
Infected B cells localize to B cell follicles in SAP-deficient mice, but fail to form GCs and do not replicate. (A, B) Representative spleen section from C57Bl6 and SAP−/− mice infected intranasally with 1,000 pfu of MHV68-H2bYFP and harvested at 16 days post-infection. Sections were stained with anti-IgD to detect naïve B cells in the mantle zone surrounding germinal centers, anti-GFP to enhance the signal from H2bYFP expressed in infected splenocytes, and anti-FDC-M1 to detect the follicular dendritic cell network. (C, D) To detect proliferating cells, mice were infected intranasally with 1,000 pfu of MHV68-H2bYFP. At day 16 post-infection, mice were injected IP with 100 mg of EdU and spleens were harvested 5 hours after treatment. Sections were stained with anti-IgD and anti-GFP as in (A), and EdU was detected with Alexa 555 conjugated azide. The approximate locations of the light zone (solid line oval) and dark zone (dotted line oval) in the MHV68 infected C57Bl/6 spleen section are shown in panel C. Scale bars represent 100 um.

To determine the physical location of MHV68 infected splenocytes, sections from spleens harvested at 16 days post-infection were analyzed by immunofluorescence microscopy. As shown in figure 4A, H2bYFP-positive B cells can be easily detected in well-delineated germinal centers in infected C57Bl/6 mice. However, in SAP-deficient mice, only small clusters of H2YFP-positive B cells could be found (Fig. 4B). In the SAP-deficient mice these clusters of infected cells were located in B cell follicles and were always associated with the follicular dendritic cell network. There was also a slight down-regulation of IgD in some follicles that contained H2bYFP-positive cells. However, it is important to note that these clusters of infected cells were extremely rare - multiple sections had to be analyzed to find even a single cluster, whereas in wild type mice numerous MHV68 infected germinal centers could were observed in each section.

Because a substantial percentage of H2bYFP-positive cells exhibited a GC phenotype (Fig. 2C,D), coupled with the observation that small clusters of infected cells could be found in sites where normal GC formation occurs (Fig. 4B), we hypothesized that the reduced number of MHV68 infected B cells in SAP-deficient mice was due to the inability of these cells to proliferate. To see if infected B cells proliferated in SAP-deficient mice, mice were injected intraperitoneally with EdU at 16 days post-infection and spleens were harvested 5 hours later. As shown in figure 4C, numerous proliferating H2bYFP-positive B cells were found in GCs of wild type C57Bl/6 mice. We have previously shown that there are 2 distinct populations of virus infected/YFP+ cells in infected germinal centers – those that are YFP-bright, which correspond to the non-proliferating infected centrocytes, and those that are YFP-dim, which correspond to the rapidly proliferating infected centroblasts [31]. EdU preferentially stains centroblasts, which are located in the dark zone (denoted by the dotted oval in Fig. 4). However, in SAP-deficient mice, infected cells incorporated EdU poorly - indicating that they were much less proliferation than infected B cells in wild type mice (Fig. 4D). Taken together, this data indicates that although B cells in SAP-deficient mice latently infected with MHV68 can acquire a GC phenotype and are physically located in B cell follicles, they are unable to proliferate. This inability to proliferate results in fewer infected B cells in SAP-deficient mice, and suggests that MHV68 infected GC B cells are dependent on signals from T_FH_ cells to drive expansion of the latently infected B cell population. Alternatively, it is formally possible that entry and/or exit of MHV68 infected SAP-deficient B cells from B cell follicles is significantly different than virus infected SAP-sufficient B cells – resulting in an under-estimation of infected B cell proliferation in SAP-deficient mice.

### SAP Expression in CD4 T Cells Is Required for Efficient Establishment of MHV68 Latency in B Cells

**Figure 5:**
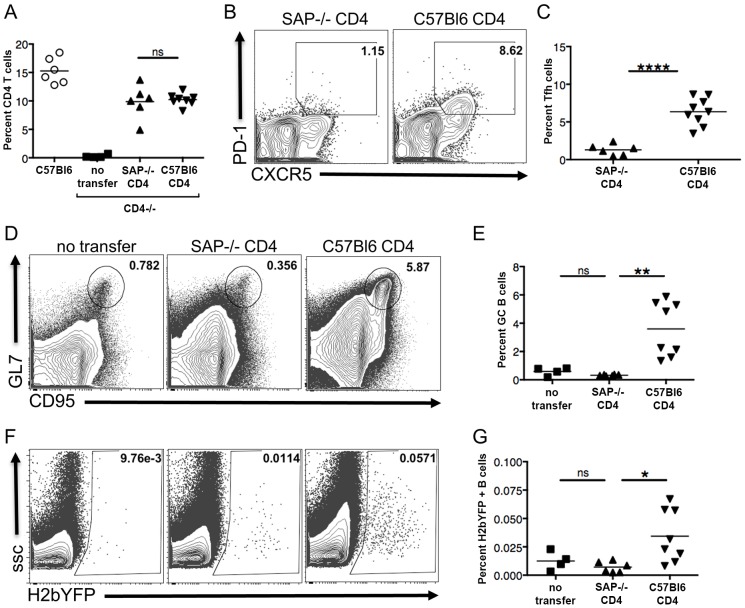
SAP expression in CD4 T cells is required for efficient establishment of MHV68 latency in B cells. CD4+ T cells from either SAP-deficient or C57Bl/6 mice were adoptively transferred into CD4−/− mice. One week after transfer, mice were infected intranasally with 1,000 pfu of MHV68-H2bYFP, and spleens were harvested 16–18 days post-infection. (A) Quantitation of the percentage of CD4+ T cells in mice that received either SAP-deficient or C57Bl/6 CD4+ T cells. Each symbol represents a single animal, and the horizontal bar represents the mean. The differences between the frequency of virus CD4+ T cells was not statistically different between infected C57Bl/6 and SAP−/− mice (p = 0.76). (B & C) SAP-deficient CD4 T cells do not differentiate into T_FH_ cells after infection. Flow cytometry plots were gated on CD4+ cells, and T_FH_ cells were defined are CXCR5^HI^, PD-1^HI^ (representative flow plots are shown in panel B). (C) Quantitation of the percentage of CD4 T cells that had differentiated into T_FH_ cells. Each symbol represents an individual infected mouse, and the horizontal line represents the mean frequency of virus infected B cells. ****, p<0.0001. (D) SAP-deficient CD4 T cells did not rescue the inability of CD4−/− mice to generate germinal center B cells. Flow cytometry plots were gated on CD3−, B220+ cells, and germinal center B cells were defined as CD95^HI^, GL7^HI^. (E) Quantitation of the percentage of germinal center B cells. Each symbol represents an individual infected mouse, and the horizontal line represents the mean frequency of virus infected B cells. There was no statistically significant difference between the frequency of T_FH_ cells between the no transfer group and those receiving SAP−/− CD4+ T cells (ns, p = 0.05), while there was a statistically significant difference between mice receiving SAP−/− CD4+ T cells vs those receiving C57B/6 CD4+ T cells (**, p = 0.0014). (F & G) MHV68-H2bYFP establishes B cell infection more efficiently in mice that received wild type CD4 T cells. Flow cytometry plots were gate on CD3−, B220+ cells (representative flow plots are shown in panel F). (G) Quantitation of the percentage of B cells that were infected as determined by H2bYFP expression. Results shown are from 2 independent experiments with 2–5 mice per group. Each symbol represents an individual infected mouse, and the horizontal line represents the mean frequency of virus infected B cells. There was no statistically significant difference between the frequency of infected B cells between the no transfer group and those receiving SAP−/− CD4+ T cells (ns, p = 0.22), while there was a statistically significant difference between mice receiving SAP−/− CD4+ T cells vs those receiving C57B/6 CD4+ T cells (*, p = 0.0151).

While the analyses of MHV68 infection in SAP-deficient mice suggested a key role for CD4 T cells in establishment of infection by MHV68, we could not rule out the possibility that the lack of efficient infection was due to an unforeseen role that SAP-deficient CD8 T cells were playing in controlling infection. Although SAP-deficient murine CD8 T cells have been shown to be unable to recognize B cell targets [38], they are more highly activated during infection with MHV68 [35], [36], and other factors such as increased cytokine production may play a role in controlling infection. To specifically address the role of CD4 T cells in the establishment and expansion of MHV68 latency, we performed adoptive transfers of either wild type or SAP-deficient CD4 T cells into CD4 knockout mice such that only CD4 T cells would be deficient in SAP expression. One week after transfer, mice were infected with 1,000 pfu of MHV68-H2bYFP, and spleens were harvested 16–18 days post-infection. As shown in figure 5A, there was no difference in the percentage of CD4 T cells in splenocytes harvested from mice that had received either SAP-deficient or C57Bl6 CD4 T cells, although the levels were slightly lower than that seen in wild type C57Bl/6 mice. Consistent with the critical role of SAP in maintenance of the pool of T_FH_ cells during the GC response, there were significantly more T_FH_ cells in mice that received C57Bl/6 CD4 T cells than mice that received SAP-deficient CD4 T cells (Fig. 5B,C). Analysis of the GC B cell populations showed that SAP-deficient CD4 T cells were unable to induce differentiation of GC B cells and that the GC response was not significantly different than that seen CD4−/− mice that received no CD4 T cells (Fig. 5D,E). However, mice that received C57Bl6 CD4 T cells exhibited a robust germinal center response following MHV68 infection (Fig. 5D,E). Importantly, this increase in the germinal center response in mice that received wild type CD4 T cells corresponded with a significant increase in the percentage of MHV68 infected (H2bYFP+) B cells compared to mice that received SAP-deficient CD4 T cells (Fig. 5F,G). Furthermore, consistent with the failure to mount an effective GC response, adoptive transfer of SAP-deficient CD4 T cells into CD4-deficient mice had no impact on the percentage of MHV68 infected (H2bYFP+) B cells compared to CD4−/− mice that received no CD4 T cells. Taken together, this data shows that SAP expression in CD4 T cells is required for efficient establishment of splenic latency by MHV68.

### Inhibiting Differentiation of Naïve CD4 T Cells into T_FH_ Results in Reduced MHV68 B Cell Latency

**Figure 6:**
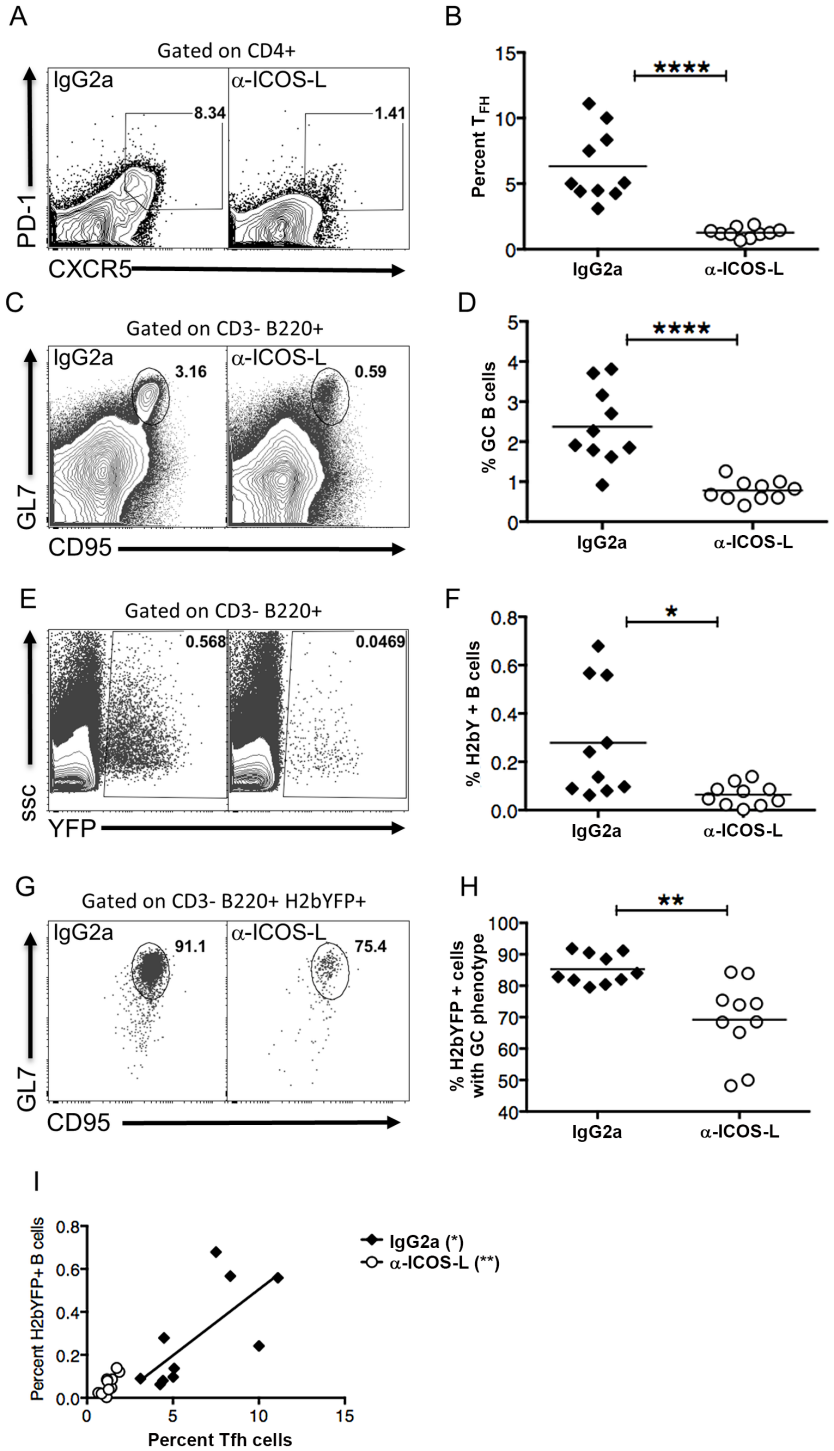
Differentiation of T_FH_ cells is required for efficient establishment of MHV68 latency in B cells. Mice were treated with 100-ICOS-l or isotype control antibody (IgG2a) beginning 2 days prior to infection and continuing every other day until spleens were harvested. Mice were infected intranasally with 1,000 pfu of MHV68-H2bYFP and splenocytes were harvested 16 days post-infection. (A & B) Anti-ICOS-L treatment inhibits differentiation of naïve CD4 T cells into T_FH_ cells. Flow plots were gated on CD4+ cells, and T_FH_ cells are defined as CXCR5^HI^, PD-1^HI^ (representative flow plots are shown in panel A). (B) Quantitation of the percentage of CD4 T cells that had a T_FH_ phenotype after treatment with either isotype control antibody (IgG2a) or anti-ICOS-L. Each symbol represents an individual infected mouse, and the horizontal line represents the mean frequency of virus infected B cells. ****, p<0.0001. (C & D) Anti-ICOS-L treatment inhibits germinal center B cell formation. Flow cytometry plots were gated on CD3−, B220+ cells, and GC B cells are defined as CD95^HI^ GL7^HI^ (representative flow plots are shown in panel C). (D) Quantitation of the percentage germinal center B cells. Each symbol represents an individual infected mouse, and the horizontal line represents the mean frequency of virus infected B cells. ****, p<0.0001. (E & F) Inhibiting T_FH_ differentiation results in decreased MHV68-H2bYFP B cell infection. Flow plots are gated on CD3−, B220+ cells (representative flow plots are shown in panel C). (F) Quantitation of the percentage of B cells that are infected as determined by H2bYFP expression. Each symbol represents an individual infected mouse, and the horizontal line represents the mean frequency of virus infected B cells. *, p = 0.0109. (G & H) Inhibition of T_FH_ differentiation results in a reduced percentage of infected B cells that have a germinal center phenotype. Flow plots were gated on CD3−, B220+, H2bYFP+, and cells with a GC phenotype were defined as being CD95^HI^, GL7^HI^ (representative flow plots are shown in panel G). **, p = 0.0012. (H) Quantitation of the percentage of infected B cells that have a GC phenotype. **, p = 0.0012. (I) There is direct relationship between the magnitude of the TFH response and the percentage of splenic B cells that are MHV68 infected (IgG2a treatment, p = 0.0209, r^2^ = 0.5070; anti-ICOS-L, p = 0.0058, r^2^ = 0.6341). Results shown are from 2 independent experiments with 5 mice per group. Each symbol in panels B, D, F, H and I represent an individual animal, and the horizontal bars represent the mean.

Since SAP expression in CD4 T cells was shown to be critical for establishment of splenic latency, this suggested that infected B cells required signals from T_FH_ cells for proliferation. Differentiation of naïve CD4 T cells into T_FH_ is mediated by signaling through ICOS, resulting in up-regulation of Bcl6 [28]. Differentiation of T_FH_ cells can be blocked with antibodies that disrupt this signaling, resulting in reduced GC formation [28]. To determine if T_FH_ differentiation is required for efficient infection of MHV68, C57Bl/6 mice were treated with either anti-ICOS-L antibodies or an isotype control antibody. Notably, treatment with anti-ICOS-L resulted in a significant reduction in the T_FH_ population compared to animals treated with the isotype control IgG2a antibody (Fig. 6A,B). Accordingly, this decrease resulted in a significant decrease in the overall percentage of GC B cells (Fig. 6C,D). Importantly, inhibiting differentiation of T_FH_ cells also resulted in a significant decrease in the percentage of MHV68 infected (H2bYFP+) B cells (Fig. 6E,F). There was also a significant decrease in the percentage of H2bYFP+ B cells that had a GC phenotype, although ca. 65% still were able to acquire a GC phenotype (Fig. 6G,H). Importantly, there was a significant correlation between the percentage of CD4 T cells that had a T_FH_ phenotype and the percentage of virus infected B cells (H2bYFP+) in both the anti-ICOS-L and isotype control treated groups (Fig. 6I). Taken together, this data suggests that MHV68 infected B cells require signals from T_FH_ cells for expansion of the pool of latently infected B cells during the germinal center reaction. In the absence of these signals, there is a significant reduction in the number of MHV68 latently infected B cells.

## Discussion

Transit of gammaherpesvirus infected B cells through the germinal center reaction is thought to be critical for gaining access to memory B cells. This allows gammaherpesviruses to remain latent in a quiescent, long-lived pool of cells. Determining what role gammaherpesviruses play in gaining access to the germinal center reaction, as well as what host determinants are required for this to occur, is essential to gaining an understanding of how these viruses establish and maintain a chronic infection.

In this manuscript, we describe a critical role for CD4 T cells in the establishment and initial expansion of MHV68 infection in B cells. Specifically, differentiation of naïve T cells into T_FH_ cells is critical for providing helper functions to GC B cells infected with MHV68. In the absence of T_FH_ cells, MHV68 infected GC B cells are unable to proliferate, resulting in reduced viral load during the onset of latency. Because SAP is required for formation of stable conjugates of CD4 T and B cells, MHV68 infected B cells in SAP-deficient mice do not receive sufficient CD4 T cell help and fail to proliferate. This inability to proliferate results in a lower viral load, and indicates that the reason MHV68 does not induce a fatal infectious mononucleosis in these mice is because the virus is unable to efficiently drive B cell proliferation *in vivo* in the absence of CD4 help.

This requirement for T_FH_ cells seems to be in direct contrast with EBV, which is thought to play a more active role in driving B cells though the germinal center reaction to gain access to the memory pool. EBV encodes proteins thought to activate and drive naïve B cells through the GC response, bypassing the requirement for T_FH_ cells for proliferation of infected cells. This eliminates the requirement for SAP expression in CD4 T cells, resulting in a lymphoproliferation in the absence of SAP expression. In the case of EBV infection, the resulting proliferation of infected cells cannot be controlled by SAP-deficient CD8 T cells.

We do not know if the requirement for T_FH_ help in establishing latency is conserved among rhadinoviruses. However, if this requirement is conserved, this may explain why XLP patients appear to be more susceptible to EBV infection than to HHV-8. Since the primary site of HHV-8 infection is unknown, very little is known about the early events during HHV-8 infection and whether or not the virus plays any role in driving infected cells through the GC reaction. However, analysis of cells derived from HHV-8 tumors suggests that unlike EBV, at least some HHV-8 infected cells are not derived from the germinal center pathway. While EBV infected Reed-Sternberg cells in Hodgkin's lymphoma [39] as well as Burkitt's lymphoma cells [40] display levels of hypermutation similar to that of germinal center and memory B cells, HHV-8 induced B cell malignancies are thought to arise from either germinal center B cells or extra-follicular B cells. Lymphomas induced by HHV-8 include primary effusion lymphoma (PEL) and multicentric Castleman's disease (MCD) [15]. PEL cells are frequently co-infected with EBV, and these co-infected cells have heavily mutated immunoglobulin genes, indicative of somatic hypermutation during the GC reaction [41], [42]. However, both mutated and non-mutated immunoglobulin genes can be found among EBV-negative PEL cells, indicating that EBV-negative PEL cells can arise from extra-follicular, as well as post-germinal center B cells [41]. HHV-8 infected cells in MCD lack somatic hypermutation and are thought to be derived solely from extra-follicular B cells [43]. This data suggests that HHV-8 can infect naïve B cells, but unlike EBV, does not by default drive them through the germinal center reaction.

Although we have shown that naïve B cells, GC B cells and plasma cells infected with MHV68 can be detected, it is not clear if the virus directly infects all three cell types or if it preferentially infects naïve B cells that then can enter either the follicular or extra-follicular pathway. The reduced frequency of infected GC B cells in SAP-deficient mice may be due to the inability to proliferate in the absence of robust CD4 help, or may be a product of the reduced frequency of GC B cells available for infection in these mice. Although a substantial fraction of infected B cells have a GC phenotype in SAP-deficient mice, this does not provide clear evidence that the virus is able to drive naïve B cells to a germinal center phenotype. Initial differentiation of T_FH_ is induced by interaction with antigen presenting dendritic cells. This interaction is mediated by integrins, and is independent of SAP [6]. However, maintenance of the T_FH_ pool requires sustained interaction with B cells, and this interaction requires SAP expression in CD4 T cells [27], [28]. This initial dendritic cell mediated differentiation of T_FH_ cells may be sufficient to drive infected B cells to a GC phenotype.

While we do not know how MHV68 gains access to the pool of germinal center B cells, it is clear that infected germinal center B cells require T_FH_ cells for proliferation and expansion of the pool of latently infected cells. It will be of interest to determine what role, if any, the virus plays in survival of B cells exiting the germinal center reaction as well as whether or not the virus influences the fate of post-germinal center B cells towards the memory B cell pool or the long lived plasma cell pool. Another unresolved question is whether or not MHV68 resides in virus specific B cells. Since T_FH_ cells provide helper functions to B cells through cognate interactions, this would suggest that infected B cells are antigen selected. However, we cannot rule out the possibility that MHV68 has evolved mechanisms to bypass the requirement for cognate interactions while still utilizing T_FH_ signaling. Finally, it will be of significant interest to generate transgenic MHV68 viruses that, similar to EBV, can bypass the requirement for T_FH_ cells and may serve as better models to study XLP.

## Materials and Methods

### Ethics Statement

This study was carried out in strict accordance with the recommendations in the Guide for the Care and Use of Laboratory Animals of the National Institutes of Health. The protocol was approved by the Emory University Institutional Animal Care and Use Committee, and in accordance with established guidelines and policies at Emory University School of Medicine (Protocol Number: YER-2002245-031416GN).

### Mice, Virus and Infections

C57Bl6 and CD4−/− mice were purchased from Jackson laboratories (ME). SAP-deficient mice have been previously described [16]. MHV68-H2bYFP is a previously described transgenic virus that expresses a fusion protein consisting of histone H2B and the enhanced yellow fluorescent protein (eYFP) [31]. Mice were anesthetized with isofluorane prior to infection, and inoculated intranasally with either 1,000 pfu or 4×10^5^ pfu of virus diluted in 20 ml of complete DMEM.

### Limiting Dilution PCR Analysis

The frequency of viral genome positive cells was determined by limiting dilution PCR analysis as previously described [44], [45]. Briefly, single cell suspensions were plated out in 3 fold dilutions in 96 well plates, starting with 10^4^ splenocytes. Cells were diluted in a background of uninfected NIH3T12 cells such that the total cell number was 10^4^ for each dilution. There were 6 dilutions for each sample, each of which consisted of 12 replicates. Cells were lysed with proteinase K at 56°C for 6 hours followed by heat inactivation at 95°C for 20 minutes. Nested PCR was then performed using previously described primers [44], [46]. To ensure single copy sensitivity, DNA corresponding to 10, 1 and 0.1 copies of the plasmid pBamHIN, which contains the region of the viral genome being amplified, was spiked into a background of 10^4^ uninfected NIH3T12 cells. PCR products were resolved on a 2% agarose gel.

### Limiting Dilution *Ex Vivo* Reactivation

The frequency of cells capable of reactivating virus was determined by limiting dilution analysis as previously described [44], [46]. Single cell suspensions of splenocytes were resuspended in DMEM and in serial two-fold dilutions on indicator MEF monolayers. Well were scored for cytopathic effect 14 days after plating. To ensure that any observed cytophathic effect was due to reactivation and not pre-formed infectious virus, mechanically disrupted cells were plated out in parallel.

### Flow Cytometry

Single cell suspensions of splenocytes were resuspended in PBS containing 1% FBS and stained in the dark on ice for 20 minutes. Antibodies used were PeCy7 conjugated anti CD95, PerCP conjugated anti-CD4, PE conjugated anti-PD-1, anti-CD138, and anti-IgD (BD Biosciences); PerCP conjugated anti-CD3 and Pacific Blue conjugated anti-B220 (Biolegend); and Alexa 647 conjugated anti-GL7 (eBioscience). For CXCR5 staining, cells were stained with purified anti-CXCR5 (BD Biosciences) at 4°C for 1 hour in PBS containing 1% FBS, 1% BSA and 2% normal mouse serum (Sigma). Cells were then stained on ice for 30 minutes with biotin conjugated Affini-pure goat anti-rat (Jackson Immunoresearch) followed by staining with APC conjugated streptavidin for 30 minutes on ice.

### Tissue Section Preparation and Immunofluorescence Microscopy

Spleens from infected mice were embedded in OCT media (Sakura Finetek) and flash frozen in chilled isopentane (Fisher Scientific). Frozen sections were cut at a thickness of 5 um and stored at −80°C until needed. To detect infected cells, FITC conjugated anti-GFP (Rockland Immunochemicals) was used to enhance the signal of H2bYFP. IgD was detected using an Alexa 647 conjugated anti-IgD (BD Biosciences). Follicular dendritic cells were detected using a purified anti-FDC-M1 (BD Biosciences followed by Alexa 546 conjugated anti-rat (Invitrogen). All images were collected on an Axiovert 200M fluorescent microscope (Carl Zeiss) using Axiovision version 4.7 imaging software.

### EdU Labeling

To detect replicating splenocytes, mice were injected intra-peritoneally with 100 mg of 5′-ethynyl 2′-deoxyuridine (EdU)(Invitrogen) 5 hours before spleens were harvested and frozen for sectioning. EdU was detected with a Click-it EdU Alexa Fluor 555 Imaging kit (C10338) according to the manufacturer's protocol with the following modification. Sections were stained with fluorophore conjugated antibodies as described above, then fixed with 4% paraformaldehyde for 10 minutes at room temperature. EdU detection was then performed according to the manufacturer's protocol.

### Lymphocyte Purification and Adoptive Transfers

CD4+ T cells were purified by negative selection from C57Bl6 and SAP−/− spleens using a mouse CD4+ T cell isolation kit (Stemcell Technologies). After purification, CD4+ T cell purity was greater than 90%. For adoptive transfers, CD4−/− mice were sub-lethally irradiated with 450 rads the day before adoptive transfer. Irradiated mice received 1×10^7^ of either C57Bl6 or SAP−/− CD4 T cells by tail vein injection and rested for one week before infection with 1,000 pfu of MHV68-H2bYFP.

### Anti-ICOS-L Treatment

Anti-ICOS-L (clone HK5.3) and isotype control (rat IgG2a) antibodies were purchased from BioXcell. Mice were treated with 100 ug of either anti-ICOS-l or isotype control antibodies by tail vein injection. Treatment was started 2 days prior to infection, and continued every other day until spleens were harvested.

### Statistical Analysis

All data analysis was performed using GraphPad Prism software (GraphPad Software, Sand Diego, CA) The frequencies of viral genome positive cells and cells capable of reactivating virus were determined by non-linear regression analysis. Frequencies were obtained from non-linear regression fit where the regression line intersected 63.2%, corresponding to the frequency at which one event is predicted to be present in a population. Statistical significance was determined by a two-tailed unpaired Student's t test with a confidence level of 95%.
